# Efficacy of Flow Diverters in Managing Multiple Aneurysms: A Clinical Case Report

**DOI:** 10.15190/d.2023.20

**Published:** 2023-12-31

**Authors:** Erekle Ekvtimishvili, Rahul Yedpallikar Rajesh, Rowyna Reji Koshy, Binu Thomas Maliyil, Arsha Anil Kumar

**Affiliations:** ^1^Endovascular Neurosurgery Department, High Technology Medical Center, University Clinic, Tbilisi, Georgia; ^2^Tbilisi State Medical University, Tbilisi, Georgia; ^3^Department of Medicine, Faculty of Medicine, Tbilisi State Medical University, Tbilisi, Georgia

**Keywords:** Multiple aneurysm, Flow diverter, Cerebral aneurysms, Endovascular procedures.

## Abstract

Brain aneurysms, also known as cerebral aneurysms, are the growths of the parent artery. Based on their shape, aneurysms can be categorized as saccular or non-saccular. Several factors have been linked to multiple brain aneurysms but the most prevalent is arterial hypertension. In the treatment of cerebral aneurysms, endovascular techniques like coil embolization have essentially taken the place of surgical clipping. Brain aneurysm treatment has undergone a revolutionary change owing to flow diverters. They are now used for complex aneurysms that are unresponsive to standard therapies, in addition to unruptured lesions that were previously treated with standard surgical or coil-based endovascular procedures. Flow diversion has shown to be a more successful treatment for aneurysms than other approaches due to its reduced complication and high recovery rate. We discuss the case of a patient who arrived with headache and was subsequently diagnosed with numerous aneurysms of the left internal carotid artery. Flow diverter stenting was employed to thrombose our patient's existing aneurysms, followed by dual antiplatelet therapy (Ticagrelor and Thrombo ASS) as a post-procedural need. Despite the fact that our patient had both large and minor aneurysms impacting challenging locations, there were no complications after treatment.

## 1. Introduction

Aneurysms in the brain (Cerebral aneurysms) are the parent artery's outgrowth. The arterial bifurcation is where they primarily start^1^. Morphology allows us to categorize aneurysms as saccular or non-saccular. Though non-saccular aneurysms are often fusiform dissecting aneurysms, unconnected to vascular branching, and more commonly involve the posterior circulation, saccular aneurysms are typically found at vascular branching locations and are more common in the anterior circulation^2^. Cerebral aneurysms can be sporadically acquired. However, there is evidence of a genetic predisposition to Cerebral aneurysms as well as a familial incidence. Numerous factors have been linked to multiple brain aneurysms; the most prominent one was hypertension, but other factors such as female sex and advancing age have also been reported. Additionally, autosomal dominant polycystic kidney disease and the familial incidence of Cerebral aneurysm were linked to multiplicity^1^. Due to the increased risk of cerebral embolism and compression-induced nerve damage, rupture, and bleeding, carotid artery aneurysms should be treated. These pseudo aneurysms and aneurysms typically cause no symptoms. Moreover, patients who experience neurological abnormalities following neck injuries should be suspicious for carotid artery aneurysms^3^.

The management of cerebral aneurysms has changed over time; with the emergence of endovascular techniques such as coil embolization replacing surgical clipping. Modern advancements in therapy, like flow diverters and Woven Endo Bridge (WEB) devices, provide sophisticated options^1^. Flow Diverters are innovative stents with higher pore density (smaller pores than ordinary stents) and more elastic to fit the vessel profile (higher metal coverage), resulting in flow stagnation and gradual thrombosis inside the aneurysm. They have redefined the treatment of brain aneurysms. They are now used not just for unruptured lesions that were previously treated with traditional surgical or coil-based endovascular procedures, but also for complex aneurysms (giant, fusiform, and blister) that are resistant to conventional therapies. Due to its favourable low complication rate and high cure rate as compared to alternative procedures, flow diversion has become increasingly important in the treatment of aneurysms^4^. Here, we present the case of a patient who presented with headache and who was eventually diagnosed as having multiple aneurysms of the left internal carotid artery.

## 2. Case report

**Figure 1:**
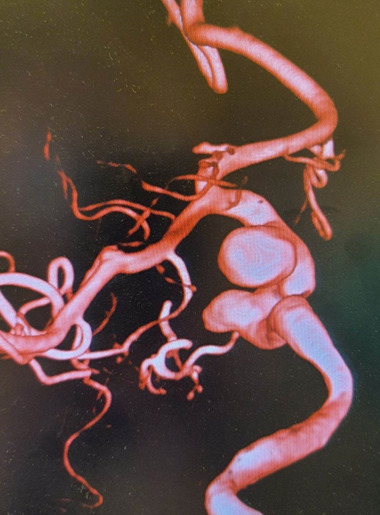
*Left internal carotid artery* MRA demonstrating multiple aneurysm affecting the ophthalmic & cavernous (C4-C6)

A 64-year-old Georgian female presented for consultation at High Technology Medical Center University Clinic with a history of transient headache for 3 months. She did not have other difficulties suchas seizures, visual impairments, respiratory symptoms or cardiovascular manifestations. No past relevant history was noted. The results of vitals and physical examinations were normal. Blood Work-up was done which was unremarkable. Imaging studies like Magnetic resonance angiography (MRA) confirmed the presence of multiple unruptured aneurysms of sizes 8mm, 4mm, and 2mm in the left internal carotid artery (Figure 1).

**Figure 2:**
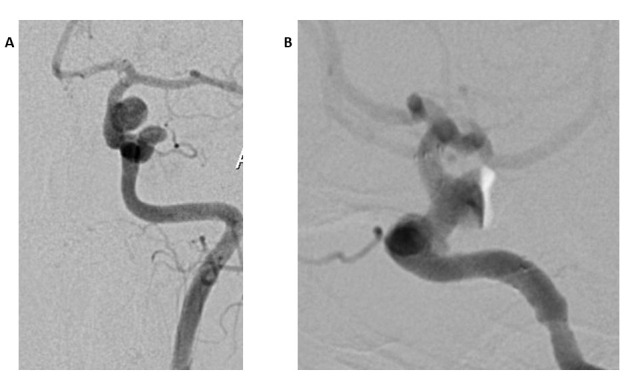
Diagnostic 2D - DSA of Left Internal Carotid of patient Demonstrating multiple aneurysm of sizes 2mm, 4mm & 8mm (A and B).

This was followed by a Digital subtraction angiography (DSA) (Figure 2).

**Figure 3:**
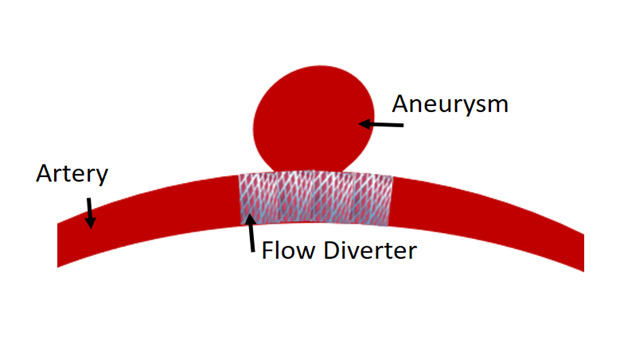
Diagram representing placement of flow diverter

Furthermore, two of these aneurysms were located in the carotid ophthalmic segment (C6) and the cavernous sinus, which were difficult areas to reach. Hence, we approached with a flow diverter stent strategy (Microvention’s FRED) without coiling to occlude all three aneurysms at the same time (Figure 3).

**Figure 4:**
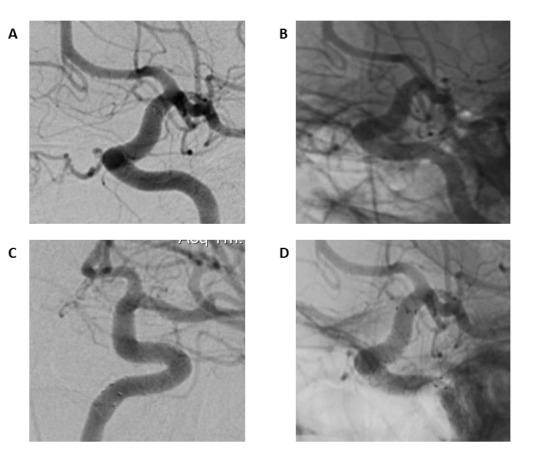
Management of the patient A and B - patient was treated with single flow diverter for aneurysms; C and D - On follow up - Control DSA was performed 12 months later, the aneurysms were seen to be thrombosed with no flow limitations.

5 days prior to the surgery, dual antiplatelet therapy was introduced. For six months after the flow diverter was placed, patient received doses of 100 mg of thrombo ASS (acetylsalicylic acid) once daily and 90mg of ticagrelor twice daily. After the six-month period, only 100 mg of thrombo ASS was administered for an additional six months. On follow-up, one year later, a control Digital subtraction angiography (DSA) was performed which indicated successful recovery (Figure 4).

## 3. Discussion

About 2% of people have cerebral aneurysms, which are primarily responsible for 80% of non-traumatic subarachnoid hemorrhages that occur when they rupture. According to autopsies, the prevalence is 1-2%, and 50-80% of them are unruptured. Unruptured cerebral aneurysms (UCAs) range from 0.3% to 4.0% in all age categories, whereas total cerebral aneurysms (including ruptured ones) range from 1.3% to 7.6%. In 10.7–34% of instances, multiple aneurysms develop. An estimated 5- 10% of cases are thought to involve unruptured aneurysms that are found by accident or that manifest as symptoms like cranial nerve palsy. Unruptured cerebral aneurysms are more often diagnosed in women than in men, with a 3:1 ratio. This is partly due to advancements inimaging technology ^1^,^5^,^6^. The primary complaint of our patient was headache which is one of the most prevalent neurological symptoms and one of the most common causes for visiting the emergency department. It affects more than half of the population at any given moment^7^. The main diagnostic tool approached by us for this case of multiple aneurysm was MR (magnetic resonance imaging (MRI) and magnetic resonance angiogram (MRA)) and (DSA) Digital subtraction angiography. With the advancement of medical imaging technologies in recent years, the identification rate of intracranial aneurysm has also increased. At the moment, Digital subtraction angiography (DSA) remains the "gold standard" for detecting these aneurysms, with a sensitivity of over 95%8. Clear observation is required from the standpoint of morphology. The relationship betweenthe tumor body and the parent artery, as well as the diameter of the tumor, can be analyzed^8^. According to Wang et al, the detection performance of aneurysms of different diameters, the detection ability of aneurysms of ≥5 mm is better than that of aneurysms of <5.0 with the use of Digital subtraction angiography (DSA)^8^. Considering the detection and size of aneurysm being large enough in our case report which were, 8mm ,4mm & 2mm, Digital subtraction angiography (DSA) has proven to be an effective diagnostic tool. Since the popularity of cerebral stenting in the early 1990s, Dual Antiplatelet Therapy (DAPT) application in endovascular aneurysm treatment has seen major breakthroughs and refinements^9^.

Hence the management of our patient was initiated with dual antiplatelet therapy (DAPT) was given 5 days prior to our operative strategy^1^. Flow diverter stenting method was used to thrombose the existing aneurysms in our patient. Followed by Dual antiplatelet therapy delivered as a post-procedural requirement which was ticagrelor for 1 month. In previous reports of carotid artery single aneurysm, clipping or clamping have been seen as an option too^3^,^7^. There are very limited data acquired about multiple aneurysm on left internal carotid artery treated with flow diverters and hence we would like to emphasize on this strategy as there were no complications associated in the case of our patient even though there were large as well as small aneurysm affecting difficult areas.

## 4. Conclusion

Multiple aneurysms in the brain cause severe symptoms that can be treated with modern interventions. The use of a flow diverter for the treatment of multiple unruptured aneurysms has been demonstrated to be effective in this case, despite the size of the aneurysms. The patient exhibits no complications following the follow-up, emphasizing the benefit of using this technique for future interventions.

## Bullet Points

• The efficacy of flow diverter treatment strategies for brain aneurysm has been documented in this evidence-based case report.

• The symptoms of multiple aneurysms in patients are often overlooked. Prompt treatment allows for a safe recovery without any complications.
